# Hyperoxia results in increased aerobic metabolism following acute brain injury

**DOI:** 10.1177/0271678X16679171

**Published:** 2016-01-01

**Authors:** Arnab Ghosh, David Highton, Christina Kolyva, Ilias Tachtsidis, Clare E Elwell, Martin Smith

**Affiliations:** 1 Neurocritical Care, University College London Hospitals, National Hospital for Neurology & Neurosurgery, London, UK; 2 Department of Medical Physics and Biomedical Engineering, University College London, London, UK; 3 University College London Hospitals National Institute for Health Research Biomedical Research Centre, London, UK

**Keywords:** Brain ischaemia, energy metabolism, mitochondria, near infrared spectroscopy, neurocritical care

## Abstract

Acute brain injury is associated with depressed aerobic metabolism. Below a critical mitochondrial pO_2_ cytochrome *c* oxidase, the terminal electron acceptor in the mitochondrial respiratory chain, fails to sustain oxidative phosphorylation. After acute brain injury, this ischaemic threshold might be shifted into apparently normal levels of tissue oxygenation. We investigated the oxygen dependency of aerobic metabolism in 16 acutely brain-injured patients using a 120-min normobaric hyperoxia challenge in the acute phase (24–72 h) post-injury and multimodal neuromonitoring, including transcranial Doppler ultrasound-measured cerebral blood flow velocity, cerebral microdialysis-derived lactate-pyruvate ratio (LPR), brain tissue pO_2_ (p_br_O_2_), and tissue oxygenation index and cytochrome *c* oxidase oxidation state (oxCCO) measured using broadband spectroscopy. Increased inspired oxygen resulted in increased p_br_O_2_ [Δp_br_O_2_ 30.9 mmHg *p* < 0.001], reduced LPR [ΔLPR −3.07 *p* = 0.015], and increased cytochrome *c* oxidase (CCO) oxidation (Δ[oxCCO] + 0.32 µM *p* < 0.001) which persisted on return-to-baseline (Δ[oxCCO] + 0.22 µM, *p* < 0.01), accompanied by a 7.5% increase in estimated cerebral metabolic rate for oxygen (*p* = 0.038). Our results are consistent with an improvement in cellular redox state, suggesting oxygen-limited metabolism above recognised ischaemic p_br_O_2_ thresholds. Diffusion limitation or mitochondrial inhibition might explain these findings. Further investigation is warranted to establish optimal oxygenation to sustain aerobic metabolism after acute brain injury.

## Introduction

The brain relies on aerobic metabolism to meet its substantial energy needs and, in health, various mechanisms ensure that oxygen (and metabolic substrate) supply is balanced to meet metabolic demand. This balance is often disturbed after acute brain injury in which cerebral hypoxia–ischaemia is a key injury mechanism associated with poor outcome, irrespective of brain injury type.^1–3^ Specific neuroprotective therapies have failed to translate into clinical benefit^4,5^ and treatment of severe acute brain injury therefore focuses on avoiding or minimising secondary cerebral hypoxia–ischaemia and consequent mitochondrial energy failure by maintaining cerebral oxygen delivery at a level that is sufficient to meet metabolic demand.^6,7^ Debate continues whether depressed aerobic metabolism, which is common following acute brain injury, predominantly reflects oxygen deprivation or a non-ischaemic metabolic crisis.^8,9^

Mitochondria exist and function normally in a near anoxic environment, facilitating a diffusion gradient for oxygen transport from the microvasculature, and offering protection from oxidant damage. Cytochrome *c* oxidase (CCO), the terminal electron acceptor in the mitochondrial respiratory chain, is responsible for reducing oxygen to water. Its low Michaelis–Menton constant (K_m_) for oxygen means that oxidative phosphorylation may continue unimpeded in isolated mitochondria with a pO_2_ less than 1 mmHg.^10^ Below a critical ischaemic threshold, CCO is reduced and, importantly, oxygen then becomes a rate limiting substrate decreasing oxidative phosphorylation.^11,12^

In health, changes of brain tissue pO_2_ within the physiological range are not believed to influence cerebral oxygen consumption^13^ but, following acute brain injury, a range of disturbances to oxygen transport and its utilisation may complicate the relationship between pO_2_ and metabolism. Classical ischaemia describes a situation of insufficient oxygen delivery, and therefore of maximal extraction of oxygen from haemoglobin, and is characterized by a combination of large oxygen extraction fraction (OEF) measured by positron emission tomography (PET), cerebral oligaemia, and falling cerebral metabolic rate for oxygen (CMRO_2_).^14^ While prevalent early after acute brain injury, this picture is less common beyond the immediate period of injury after stroke and traumatic brain injury (TBI).^15^ Metabolic dysfunction has been identified in the presence of apparently acceptable tissue oxygenation, where both diffusion limited oxygen transport and mitochondrial dysfunction have been implicated as alternative forms of restriction to oxidative metabolism in the presence of a normal interstitial tissue pO_2_ or OEF.^16^

Multimodal neuromonitoring with brain tissue pO_2_ (p_br_O_2_) and cerebral microdialysis-derived lactate:pyruvate ratio (LPR) have enabled investigation of the relationship between oxygen delivery, cerebral tissue oxygenation, and cellular redox status in vivo following TBI,^16^ aneurysmal subarachnoid haemorrhage (SAH),^17^ and intracerebral haemorrhage (ICH).^18,19^ Clinical therapy protocols guided by changes in p_br_O_2_ seek to maintain oxygen delivery and availability above a ‘critical’ pO_2_ threshold for anaerobic metabolism.^20^ Although overt ischaemia and anaerobic metabolism has typically been described when p_br_O_2_ falls below 10 mmHg, normobaric hyperoxia and hyperbaric hyperoxia may improve LPR and CMRO_2_ after TBI in the presence of p_br_O_2_ values that are within or above the normal physiological range.^21–23^ Vespa et al.^16^ demonstrated metabolic dysfunction without classical ischaemia after TBI based on observation of elevated LPR and PET-derived OEF > 0.75. Others have described a similar picture of metabolic dysfunction ‘without hypoxia’ in SAH and ICH.^17^ However, it is difficult to entirely rule out hypoxia as a cause of such observations because of the absence of a subcellular marker of oxygenation in these studies. Diffusion limitation or an altered mitochondrial ischaemic threshold could equally explain these findings. While CCO oxidation status reflects the activity of the respiratory chain, it is also dependent on metabolic substrate supply, ATP, oxygen, and mediators which modify the K_m_ for oxygen such as nitric oxide.^24^ Understanding the changes in CCO oxidation status may therefore be a useful adjunct for the in-vivo investigation of diffusion limitation and mitochondrial dysfunction after acute brain injury.

We have developed an in-house optical technique, incorporating hybrid spatially resolved broadband and frequency domain near infrared spectroscopy (NIRS), optimised for the measurement of the oxidation state of CCO [oxCCO] in adult brain-injured patients.^25^ Spatially resolved cerebral tissue oxygen saturation, also called the tissue oxygenation index (TOI), in association with concentrations of oxyhaemoglobin ([HbO_2_]), deoxyhaemoglobin ([HHb]) and [oxCCO] may be used to investigate oxygenation of both the microvasculature and mitochondria.^12^ A comprehensive multimodal neuromonitoring array, including p_br_O_2_, microdialysis, transcranial Doppler-measured cerebral blood flow velocity and NIRS therefore covers the entire oxygen cascade from the microvasculature (TOI, HbO_2_, HHb) through the tissue interstitium (p_br_O_2_) to the mitochrondria ([oxCCO]), and has potential to predict cellular redox status (microdialysis LPR, [oxCCO]) and CMRO_2_ changes estimated using NIRS and transcranial Doppler,^26^ and might therefore differentiate between diffusion limited oxygen transport and mitochondrial dysfunction.

The aim of this study was to investigate the oxygen dependence of mitochondrial metabolism in vivo following acute brain injury. We hypothesised that normobaric hyperoxia-induced increases in cerebral oxygen availability would lead to an increase in CCO oxidation and reduction in microdialysate LPR, suggesting oxygen-limited mitochondrial oxidative metabolism at baseline.

## Materials and methods

### Study participants and protocol

**Figure 1.**
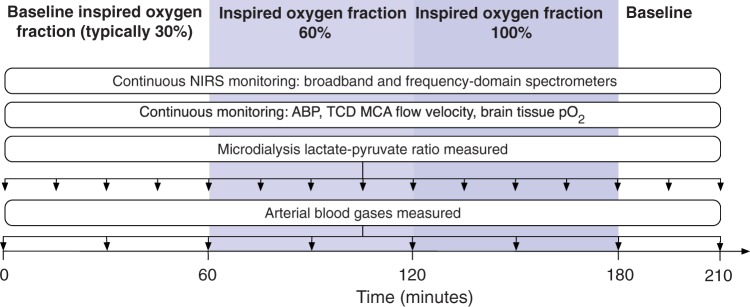
Normobaric hyperoxia protocol and measured variables.

After approval by the Research Ethics Committee of the National Hospital for Neurology and Neurosurgery and Institute of Neurology (04/Q0512/67) and representative consent, recordings were carried out in 16 sedated, mechanically ventilated acute brain injury patients requiring invasive neuromonitoring to guide clinical management on the neurocritical care unit. This was performed in accordance with the Declaration of Helsinki. Inclusion criteria also included baseline inspired fraction of oxygen (FiO_2_) less than 0.5. The patients were subject to a normobaric hyperoxia protocol which consisted of a 60-min epoch of baseline recording followed by two 60-min epochs in which FiO_2_ was increased first to 0.6 and then to 1.0, and a final 30-min epoch during which FiO_2_ was returned to baseline values (Figure 1).

### Monitored parameters

Systemic physiological monitoring included invasive arterial blood pressure (ABP) and pulse oximetry (SpO_2_) measured continuously, and measurement of arterial blood gases (ABGs), including carbon dioxide and oxygen partial pressures (paCO_2_ and paO_2_, respectively). Middle cerebral artery blood flow velocity (Vmca) was measured using transcranial Doppler ultrasonography (DWL Doppler Box, Compumedics, Singen, Germany). Invasive cerebral monitoring comprised p_br_O_2_ (Licox, Integra Neurosciences, Plainsboro, USA) and measurement of LPR by cerebral microdialysis (M Dialysis AB, Stockholm, Sweden), with catheters implanted via a cranial access device (Technicam, Newton Abbot UK or Licox IP2, Integra Neurosciences) or surgically at time of craniotomy. In accordance with consensus guidelines, catheters were placed in peri-lesional tissue in patients with focal TBI or ICH, in the right frontal lobe in patients with diffuse TBI, or tissue thought to be at risk of ischaemia from vasospasm in patients with aneurysmal SAH.^27^ All non-invasive cerebral monitoring was conducted ipsilateral to the invasive monitoring.

### NIRS instrumentation and processing

The NIRS apparatus used in this study has been described in detail elsewhere.^28^ In brief it comprises two components – a multidistance broadband spectrometer and a multidistance frequency domain spectrometer. Chromophore concentration was derived from the broadband spectroscopy component that incorporates a 50 W halogen light source and lens-based spectrograph based on a charge-coupled device camera (PIXIS 512f, Princeton instruments) using the UCLn algorithm.^29^ Measurements were made simultaneously at four source-detector separations (20/25/30/35 mm). The broadband spectrometer-derived concentrations of HbO_2_, HHb and oxCCO from the 35 mm separation are reported here as we have previously shown that this source-detector separation has the highest brain-specificity, particularly for the measurement of oxCCO.^25^ The frequency domain component of the system utilises an OxyPlexTS device (ISS Inc., Champaign, IL, USA) modified with diodes emitting light at four wavelengths (690, 750, 790 and 850 nm), and was used to derive the absolute optical absorption and reduced scattering coefficients (µa and µs, respectively) as previously described,^28^ and derivation of the differential pathlength factor using the diffusion approximation.^30^ In this study, we report µs recorded at 790 nm. An individual differential pathlength factor was calculated for each patient, based on the µa and µs measured by the frequency domain spectrometer during the initial minute of recording of the baseline epoch. The TOI – defined as [HbO_2_]/([HbO_2_]+[HHb]) – was calculated using spatially resolved spectroscopy.^31^ The 740 nm–900 nm wavelength range was used to resolve for HbO_2_, HHb, and water, and TOI calculated using individual scattering values measured with the frequency domain system.^25^

NIRS data analysis was performed in Matlab 2010b (Mathworks, Natick, MA). Differential concentrations of HbO_2_, HHb and oxCCO (Δ[HbO_2_], Δ[HHb] and Δ[oxCCO], respectively) were calculated using the UCLn algorithm.^28–30^ Changes in total haemoglobin concentration (Δ[HbT]) were calculated as Δ[HbO_2_] + Δ[HHb] and in haemoglobin difference concentration (Δ[HbDiff]) as Δ[HbO_2_] – Δ[HHb].

### Data processing

After manual identification and linear interpolation to remove NIRS signal artefacts, mean values for each monitored variable were calculated for individual epochs for each patient. The continuously monitored systemic and cerebral variables (including NIRS) were synchronized, and a mean value from a period comprising ≥ 50% of the epoch which was free from noise was used for analysis. For intermittently sampled variables (i.e. ABGs and microdialysate LPR), the mean of all readings per epoch (minimum two per epoch) was used as the summary variable for that epoch.

Relative estimated changes in CMRO_2_ (rCMRO_2_) were estimated for the return-to-baseline epoch compared to the baseline epoch using the NIRS Fick equation (equation (1)) described by Roche-Labarbe et al.^32^

(1) $$\text{rCMR}{\text{o}}_{2}=\frac{\text{Vmca}}{\text{Vmc}{\text{a}}_{0}}.(\frac{\text{Sp}{\text{O}}_{2}-\text{TOI}}{\text{Sp}{\text{O}}_{20}-\text{TO}{\text{I}}_{0}})$$

### Statistical analysis

We used GLIMMPSE, a validated model for power calculation in linear mixed models,^33^ to conduct a sample size calculation. Assuming an Δ[oxCCO] standard deviation of 0.2 µM in each epoch, a total of 16 patients are required to provide a power of 90% in detecting Δ[oxCCO] changes of + 0.1, + 0.2 and +0.05 µM during the FiO_2_=0.6, FiO_2_=1.0 and return-to-baseline epochs.

Statistical analyses were carried out in R.^34^ Parameters of interest were analysed using a mixed effects model,^35^ modelling individual subjects as random and epochs as fixed effects. The significance of the fixed epoch effect for each variable (i.e. the probability that the variable was the same across all four epochs) was then estimated using the Likelihood Ratio Test, comparing the mixed effects model to a null model comprising only random effects. In variables with an epoch effect probability of <0.05, subsequent pairwise comparison between baseline and subsequent FiO_2_ epochs (0.6 and 1.0 and return-to-baseline), was performed using Bonferroni-corrected Wilcoxon signed-rank tests. The Hodges–Lehman estimate was used to calculate the (pseudo)median and per-epoch 95% confidence intervals. Relative changes in CMRO_2_ were similarly treated, but no Bonferonni correction was applied since only the baseline and return-to-baseline epochs were compared. All data are expressed as (pseudo)median (95% confidence interval) unless otherwise stated. A Spearman correlation was used to assess the relationship between baseline p_br_O_2_ and LPR and the ΔLPR response to normobaric hyperoxia. Statistical significance was inferred at *p* < 0.05.

## Results

**Figure 2.**
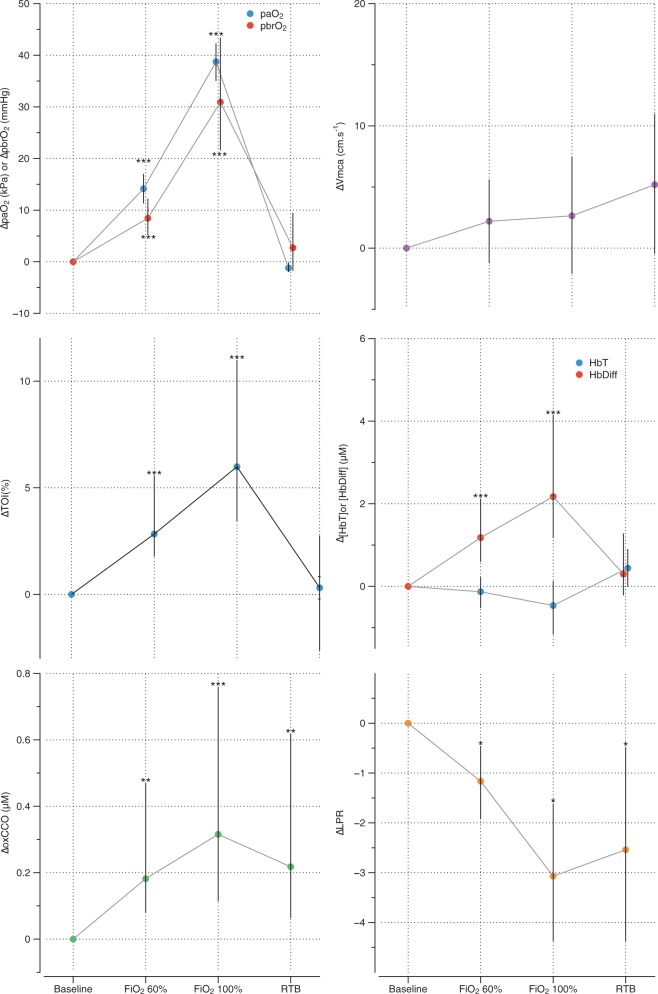
Changes in markers of cerebral oxygen delivery and aerobic metabolism during normobaric hyperoxia showing (pseudo)median changes and 95% confidence interval error bars. *p < 0.05; **p < 0.01; ***p < 0.001.

The full study protocol was completed in all 16 patients. Patient characteristics are shown in Table 1. Technical failure resulted in the loss of ABP and p_br_O_2_ recordings for one patient but this patient was included in all analyses, excluding these parameters. Baseline levels for the physiological variables are shown in Table 2, the epoch effect for each variable in Table 3, and changes in measured variables in Table 4 and Figure 2.

**Table 1.** Demographic data.

Age (years)	46.5 (39.3–51.5)
Sex	6 male, 10 female
Primary diagnosis	TBI 7 SAH 8 ICH 1
Time to study (hours post injury)	36 (25.5–45)
Admission GCS	7 (4–9)

Note: Data expressed as median with IQR.

GCS: Glasgow coma score; ICH: intracerebral haemorrhage; SAH: subarachnoid haemorrhage; TBI: traumatic brain injury.

**Table 2.** Physiological & optical variables at baseline.

Variable	Baseline value (IQR)
FiO_2_	0.325 (0.28–0.35)
paO_2_ (kPa)	15.7 (12.5–18.0)
paCO_2_ (kPa)	4.85 (4.65–4.97)
SpO_2_ (%)	99 (98–99)
MAP (mmHg)	91.5 (83.2–96.8)
Vmca (cm.s^−1^)	52.1 (48.7–71.8)
pbrO_2_ (mmHg)	17.5 (12.0–24.4)
Microdialysate LPR	25.3 (23.5–33.5)
TOI (%)	72.8 (65.5–77.0)
DPF	9.33 (6.75–10.8)
µs (cm^−1^)	11.1 (6.76–12.4)

DPF: differential pathlength factor; FiO_2_: inspired oxygen fraction; IQR: inter-quartile range; MAP: mean arterial blood pressure; LPR: lactate:pyruvate ratio; paO_2_: arterial pO_2_; paCO_2_: arterial pCO_2_; pbrO_2_, brain tissue pO_2_; TOI: tissue oxygenation index; SpO_2_: arterial oxygen saturation; µs: optical reduced scattering coefficient; Vmca: middle cerebral artery blood flow velocity.

**Table 3.** Epoch effects from likelihood ratio test.

Variable	Chi-squared	*p*
paO_2_	171	<0.001
pCO_2_	5.63	0.131
SpO_2_	64.8	<0.001
LPR	9.28	0.026
pbrO_2_	44.6	<0.001
Vmca	8.31	0.04
HbDiff	27.0	<0.001
HbT	4.5	0.21
oxCCO	15.1	0.002
TOI	22.3	<0.001
µs	1.06	0.787

HbDiff: haemoglobin concentration difference; HbT: total haemoglobin concentration; LPR: lactate:pyruvate ratio; oxCCO: cytochrome *c* oxidation state; paO_2_: arterial pO_2_; paCO_2_: arterial pCO_2_; pbrO_2_: brain tissue pO_2_; TOI: tissue oxygenation index; SpO_2_: arterial oxygen saturation; µs: optical reduced scattering coefficient; Vmca: middle cerebral artery blood flow velocity.

**Table 4.** Changes from baseline for measured variables data presented as (pseudo)median (95% confidence interval.

	Epoch		
	FiO_2_ 0.6	FiO_2_ 1.0	Return-to-baseline
Δ[HbDiff] (µM)	1.18 (0.59–2.12)	2.17 (1.17–4.13)	0.30 (−0.22–1.28)
Δ[HbT] (µM)	−0.13 (−0.52–0.22)	−0.46 (−1.16–0.13)	0.44 (−0.01–0.90)
Δ[oxCCO] (µM)	0.18 (0.08–0.47)	0.32 (0.11–0.76)	0.22 (0.06–0.62)
ΔTOI (%)	2.8 (1.8–5.6)	6.0 (3.4–10.9)	0.31 (−2.6–2.8)
Relative CMRO_2_ (%)	–	–	107.5 (100.3–119.0)
Δ LPR	−1.16 (−1.93–−0.455)	−3.07 (−4.38–−1.61)	−2.54 (−4.38–−0.475)
Δ pbrO_2_ (mmHg)	8.44 (5.19–12.2)	30.9 (21.6–43.4)	2.72 (−1.76–9.46)
Δ MAP (mmHg)	1.19 (−2.32–4.92)	1.48 (−3.73–8.8)	−0.56 (−7.83–7.38)
Δ pCO_2_ (kPa)	0.15 (0.0333–0.258)	0.114 (-0.05–0.258)	0.203 (−0.1–0.425)
Δ paO_2_ (kPa)	14.1 (11.3–17)	38.7 (35–42.3)	−1.21 (−1.99–−0.1)
Δ SpO_2_ (%)	1.5 (1.00–2.00)	1.5 (1.01–2.00)	−0.831 (−1.16–−0.778)
Vmca (cm.s^−1^)	2.19 (−1.23–5.62)	2.64 (−2.09–7.47)	5.19 (−0.45–11)
µs (cm^−1^)	0.0168 (−0.201–0.173)	−0.0201 (−0.394–0.297)	0.0785 (−0.423–0.354)

FiO_2_: inspired oxygen fraction; CMRO_2_: cerebral metabolic rate for oxygen; HbDiff: haemoglobin concentration difference; HbT: total haemoglobin concentration; LPR: lactate:pyruvate ratio; MAP: mean arterial blood pressure; oxCCO: cytochrome *c* oxidation state; paO2: arterial pO_2_; paCO2: arterial pCO2; pbrO_2_: brain tissue pO_2_; TOI: tissue oxygenation index; SpO_2_: arterial oxygen saturation; µs: optical reduced scattering coefficient; Vmca: middle cerebral artery blood flow velocity

Normobaric hyperoxia was associated with statistically significant increases in paO_2_, SpO_2_ and p_br_O_2_, but there was no change in paCO_2_ during the study. While a significant overall epoch effect for Vmca was observed, post hoc testing identified no single epoch difference from baseline. Normobaric hyperoxia was also associated with statistically significant increases in Δ[oxCCO] and reductions in microdialysate LPR during the 0.6 FiO_2_ (Δ[oxCCO] + 0.18, *p* < 0.01; ΔLPR −1.16, *p* < 0.01) and 1.0 FiO_2_ (Δ[oxCCO] + 0.32, *p* < 0.001; ΔLPR −3.07, *p* < 0.01) epochs. These changes persisted in to the return-to-baseline epoch (Δ[oxCCO] + 0.22 [*p* < 0.01] and ΔLPR −0.254 [*p* < 0.01]). Estimated CMRO_2_ was higher in the return-to-baseline epoch compared to the baseline epoch [ΔCMRO_2_ 107.5% of baseline (95% CI 100.3% – 119.0%, *p* = 0.039)].

There were no changes in Δ[HbT] during the study. [HbDiff] increased during the 0.6 and 1.0 FiO_2_ epochs (Δ[HbDiff] + 1.18 µM and +2.17, respectively, both *p* < 0.001), but there was no significant change during the return-to-baseline epoch compared to baseline. There was a significant increase in TOI during the 0.6 and 1.0 FiO_2_ epochs (ΔTOI 2.8% and 6.0% respectively, both *p* < 0.001), with no significant change during the return-to-baseline epoch. There were no significant changes in optical scattering measured at 790 nm (epoch effect *p* = 0.786). There was no correlation between baseline p_br_O_2_ or LPR and the ΔLPR response to normobaric hyperoxia (r = −0.04, *p* = 0.89; r = 0.01 *p* = 0.98, respectively).

## Discussion

We have demonstrated that normobaric hyperoxia-induced increase in p_br_O_2_ is associated with increased [oxCCO] and reduced LPR, suggesting a change in mitochondrial redox status and the presence of oxygen dependent metabolism above traditionally described ischaemic thresholds. Our findings are consistent with oxygen-limited metabolism in this cohort of patients with acute brain injury, and suggest the presence of either oxygen diffusion limitation or mitochondrial dysfunction and hypoxia–ischaemia despite ‘normal’ values for p_br_O_2_. Importantly, the [oxCCO] and LPR changes are sustained when FiO_2_ is returned to baseline after the period of hyperoxia, while the markers of microvascular and brain tissue oxygenation (TOI, [HbO_2_], [HHb], p_br_O_2_) return to their pre-hyperoxia values. This suggests that improvement in cellular metabolism persists beyond the immediate period of normobaric hyperoxia, a supposition supported by the elevation in estimated CMRO_2_ during the return-to-baseline FiO_2_ epoch.

Although the mean baseline p_br_O_2_ of 17.5 mmHg in our study lies within some definitions of hypoxia–ischaemia (<20 mmHg),^36^ the majority of previous studies highlight <10 mmHg as a particular risk for elevated LPR and PET markers of ischaemia.^37,38^ In our study, both epochs of the hyperoxia protocol resulted in elevation of p_br_O_2_ well into its ‘normal’ physiological range, and there was a stepwise increase in [oxCCO] and reduction in LPR as FiO_2_ was increased from 0.6 to 1.0. These findings are not consistent with classical hypoxia–ischaemia. There was also no correlation between baseline p_br_O_2_ or LPR and the change in LPR, suggesting that hypoxia/ischaemia, defined by pbrO_2_ or LPR, does not affect the brain's response to normobaric hyperoxia in this patient group. This finding is unsurprising since the LPR was consistently reduced (−3.07 95% CI −4.38–−1.61) during normobaric hyperoxia despite different baseline values for p_br_O_2_ and LPR.

Both oxygen diffusion abnormalities and mitochondrial dysfunction have been proposed as mechanisms for oxygen becoming a rate limiting substrate for metabolism.^15,16,21^ Delivery of oxygen to the mitochondria is dependent on the gradient of oxygen tension as well as the conductance of the tissues. During the study period (24–72 h after ictus), cerebral oedema and hence perivascular/cellular swelling and microvascular collapse are important factors which increase the diffusion distance from the microvasculature to mitochondria and might necessitate increased oxygen tension to sustain the rate of mitochondrial oxygen delivery. However, our findings of sustained metabolic improvement ([oxCCO], LPR, CMRO_2_) on return-to-baseline FiO_2_ and therefore baseline paO_2_, and predicted oxygenation gradients (see below), are not entirely consistent with diffusion limitation as the only pathophysiological process. They may also indicate reversal of mitochondrial dysfunction by normobaric hyperoxia. Baseline TOI was 73% in our study and this lies within a physiologically ‘normal’ range for NIRS-derived regional cerebral saturation.^39^ Assuming one-quarter of blood volume is saturated arterial blood, this predicts a venous saturation of 64% and approximate venous pO_2_ of 33 mmHg (using the calculation from Menon et al.^15^), and thus an average difference of 15.5 mmHg between venous blood (33 mmHg) and p_br_O_2_ (17.5 mmHg). Similar comparisons using PET and p_br_O_2_ have described gradients of 10 mmHg and 27 mmHg in normal and impaired brain regions,^15^ so our observations are consistent with only a moderate diffusion distance between the microvasculature and tissue interstitium. This further supports the notion that isolated diffusion limitation is not the sole mechanism implicated in oxygen becoming a rate limiting substrate for metabolism after acute brain injury.

CCO oxidation increased by 0.32 µM during a mean p_br_O_2_ change of 30.9 mmHg in the 1.0 FiO_2_ epoch, and returned to 0.22 µM during return-to-baseline FiO_2_. Although the total concentration of CCO in the adult human brain is unknown, it is approximately 5 µM in rats.^40^ The CCO changes that we observed are therefore likely to reflect an approximate 6% change in its oxidation, which is higher than that observed in healthy volunteers during increases in cerebral oxygen delivery or during functional activation,^25,28,41,42^ but equivalent to those described previously in TBI.^22^ The oxidation status of CCO is modified by both mitochondrial pO_2_ and metabolic factors (ADP, NAD:NADH), and our findings are consistent with both an increase in aerobic metabolism and/or increased mitochondrial pO_2_. Earlier studies have shown an association between cerebral oxygen delivery and CCO oxidation in healthy volunteers^25,43^ and, in animal models, with brain ATP^40^ and lactate^44^ concentrations. Likewise, the persistent CCO oxidation in the return-to-baseline epoch in our study suggests either increased aerobic metabolism (consistent with the measured LPR and estimated CMRO_2_) and/or an altered K_m_ for O_2_. It is interesting to note that nitric oxide is known to increase the K_m_ of CCO, and the proposed mechanism of action for normobaric and hyperbaric hyperoxia is the reversal of this nitric oxide effect thereby reducing the threshold at which oxygen becomes a rate limiting step in oxidative metabolism.^24^ Thus, our results could theoretically represent the breakdown of NO rather than a direct effect of elevated mitochondrial pO_2_. Hypoxia-inducible factor 1α (HIF-1α) is another major hypoxia signalling pathway which inhibits pyruvate dehydrogenase activity, a branch point controlling oxidative/anaerobic metabolism, as well as a range of glycolytic enzymes.^45^ Although these effects may also be modified by hyperoxia, it has also been suggested that a reduction in LPR (as seen in our study) is more consistent with an increase in oxidative metabolism since HIF-1α should reduce lactate while maintaining LPR.^21^ While the median reduction in LPR (−3.07) during normobaric hyperoxia in our study is not large, and of unlikely clinical significance in itself, it might reflect a small volume of ischaemic tissue within the larger tissue volume monitored by the microdialysis catheter. Furthermore, when considered within the context of the increases in aerobic metabolism shown by the other monitoring modalities during normobaric hyperoxia, it is possible that this small improvement in LPR might indicate patients with an oxygen-dependent deficit in aerobic metabolism that is amenable to treatment. Further clinical studies are required to assess the clinical relevance of such changes in LPR when interpreted in association with other monitored variables of aerobic metabolism.

Our findings are consistent with those of several previous studies. Diringer et al. found no significant change in CMRO_2_ in a PET study of normobaric hyperoxia in five TBI patients, but the small sample size precludes definitive conclusions. A pilot study by our group of eight patients with TBI found similar changes in LPR (−1.6) and Δ[oxCCO] (+0.21 µM) during normobaric hyperoxia to those we report here.^22^ Our current study builds on that pilot in three important aspects – by including a larger number of patients, using an improved NIRS apparatus with patient-specific measures of differential pathlength factor, and incorporating an estimate of CMRO_2_. Nortje et al.^21^ also demonstrated that normobaric hyperoxia in patients with TBI was associated with a similar reduction in LPR (mean LPR reduced from 34.1 to 32.5) to our current study, but PET-measured CMRO_2_ increased only in regions of interest with a reduced CMRO_2_ at baseline. However, a smaller study of eight patients with TBI, showed no improvement in LPR during 3 h of normobaric hyperoxia.^46^

Our study demonstrated an improvement in markers of aerobic metabolism during a short (120 min) graded hyperoxia challenge. Although [oxCCO], LPR and CMRO_2_ remained partially elevated following return-to-baseline FiO_2_, further assessments were not made beyond this period so the longevity of the potential metabolic benefit of hyperoxia is uncertain. It must be noted that hyperoxia has a variety of deleterious effects through generation of reactive oxygen species, induction of cytotoxic cytokines and immunosuppression, and concerns exist regarding its prolonged use. Kilgannon et al.^47^ identified an association between supranormal paO_2_ and worsened outcome following cardiac arrest, while Quintard et al.^48^ demonstrated an association between normobaric hyperoxia and increased microdialysate glutamate, a key mediator of cerebral excitotoxicity, following TBI. While reactive oxygen species are a major injury mechanism implicated following cerebral ischaemia, they may be generated both by hypoxia (excess of reductive substrate) as well as by the delivery of excessive oxygen.^49,50^ Thus, oxygen therapy must be carefully controlled after acute brain injury. Time-limited application of hyperoxia or the use of p_br_O_2_ to guide oxygen administration may limit the potential deleterious effects of unrestrained oxygen use while minimising the risk of cerebral hypoxia/ischaemia. A higher p_br_O_2_ might be warranted given the concerns with oxygen diffusion and mitochondrial inhibition 24–72 h post injury. Normobaric hyperoxia frequently results in restoration of p_br_O_2_ into what is usually considered to be a ‘normoxic’ range, and the consistent increases observed in markers of aerobic metabolism even when p_br_O_2_ is greater than the physiological range^21–23^ might suggest the need for a higher target. Future research should focus on the relevance of higher p_br_O_2_ targets and additional monitored variables that can inform oxygen therapy after acute brain injury.

Our study has several limitations. First, the individual monitoring modalities are designed to measure different aspects of the oxygen cascade and cellular bioenergetics, but each is subject to its own limitations. Although differential spectroscopy NIRS methodologies, such as the one we used to measure haemoglobin and [oxCCO] in this study, are subject to significantly more extracranial ‘contamination’ than spatially resolved spectroscopy techniques,^39^ we have previously shown that [oxCCO] is a brain-specific signal with negligible contribution from extracranial tissues.^25^ Furthermore, by measuring scattering and optical pathlength, we can place greater confidence on the accuracy of the measured change.^40^ The differential spectroscopy methodology that we used to measure changes in CCO oxidation is based on the modified Beer–Lambert law and therefore only able to quantify relative changes in chromophore concentration from an unknown baseline rather than measure absolute concentrations of oxidised and reduced CCO. Nevertheless, changes in CCO have been evaluated in animal models and shown to be a reliable measure of intracellular energy status.^40,44,51^ Microdialysate LPR is an imperfect measure of cerebral aerobic metabolism. It reflects the activity of cytosolic lactate dehydrogenase, which is in large part reflective of intracellular NADH:NAD^+^ ratio and thus related to the ability of mitochondria to produce ATP. There are therefore circumstances, including electron leak from the electron transport chain, during which LPR can be unchanged in the face of an inability of cells to generate energy.^52^ Secondly, although we placed the microdialysis catheters in ‘at risk’ tissue in line with consensus guidelines,^27^ used the same cranial access device for p_br_O_2_ monitoring, and applied the NIRS optodes over the frontal region as close as possible to the insertion site of the invasive monitors, it is likely that different tissue volumes and regions were interrogated by each device. Similarly, it is difficult to know exactly what region of interest is represented by our estimate of CMRO_2_ which is derived from Vmca (a relatively global measure of hemispheric CBF) and TOI (a regional measure of tissue oxygenation).^39^ Finally, we investigated only 16 patients with mixed pathology, but this is a larger patient cohort than many investigations in this field – for example, those cited above – and the concept of an ischaemic p_br_O_2_ threshold is relevant across all the pathologies included. Spatial heterogeneity is a limiting factor in the study of most acute brain injury types, and we have specifically targeted analysis of the injured hemisphere using a comprehensive array of monitoring modalities.

Overall, our findings highlight the difficulties in defining thresholds for hypoxia–ischaemia in the injured brain, and the potential risks to an individual of utilising generic targets to guide clinical management. Diffusion limitation and mitochondrial dysfunction may disrupt the normal relationship between OEF, p_br_O_2_, and mitochondrial redox status, and this might explain some observations of metabolic dysfunction ‘without hypoxia’ when either a diffusion barrier to oxygen is present (when p_br_O_2_ and OEF may not reflect mitochondrial pO_2_) or much higher mitochondrial pO_2_ is required to maintain ATP generation. Further studies employing multimodal monitoring, including [oxCCO], might shed further light on the exact nature of this metabolic derangement, and in the understanding of oxygen diffusion within the microvascular and intracellular environments, and oxygen utilisation. Extension of existing computational physiological models^53^ may assist in predicting oxygen diffusion gradients and utilisation, exploiting measurement of TOI, p_br_O_2_ and CCO, and should be incorporated into future studies.

## Conclusion

Standard clinical therapy following acute brain injury fundamentally aims to avoid mitochondrial hypoxia in order to minimise secondary tissue ischaemia and worse clinical outcomes. p_br_O_2_ and microdialysis-measured LPR have been used as surrogates of mitochondrial oxygen availability and its effect on mitochondrial redox status at the bedside, but clinical application and interpretation of such techniques requires clearly defined thresholds for ‘ischaemia’. Our results demonstrate an increase in oxCCO, reduction in LPR and increase in estimated CMRO_2_ during and following normobaric hyperoxia. These findings are consistent with increased aerobic metabolism at p_br_O_2_ levels higher than those typically recognised as ‘ischaemic’ thresholds. Such oxygen-limited metabolism suggests that hypoxia–ischaemia secondary to oxygen diffusion limitation or mitochondrial dysfunction might be prevalent after acute brain injury, and complicate assessment of ischaemia using measurement of p_br_O_2_ in isolation. Simultaneous measurement of microvascular, tissue and cellular oxygenation and metabolism has potential to redefine our understanding of ischaemia after acute brain injury. Measurement of the oxidation status of CCO as a bedside, continuous assessment of mitochondrial energetics over multiple regions of interest has considerable potential to guide treatment after acute brain injury.
